# Draft genome sequence and SSR mining data of *Acacia pachyceras* Schwartz

**DOI:** 10.1016/j.dib.2022.108031

**Published:** 2022-03-08

**Authors:** Nazima Habibi, Fadila Al Salameen, Muhammed Rahman, Vinod Kumar, Sami Al Amad, Anisha Shajan, Farhana Zakir, Nasreem Abdul Razzack, Waiel Hussain Tinwala

**Affiliations:** a Environment and Life Sciences Research Centre, Kuwait Institute for Scientific Research, Kuwait; b Department of Computer Science, Delhi Technological University, Delhi, India

**Keywords:** Genome survey, Molecular markers, Native plants, Whole genome sequencing, De novo assembly

## Abstract

*Acacia* tree population is declining in several countries of the world especially in the Arabian peninsula due to human-induced activities. The tree has potential medicinal and economic benefits as a source of fuel and timber. It can fix nitrogen, a significant property that assists in desert rehabilitation. However, the lack of genomic information of *Acacia pachyceras* hampers its genetic study and breeding process. We performed paired-end sequencing of *A. pachyceras* at a depth of 120X to obtain raw sequences of 108.9 GB with a per base quality >Q30. Filtered raw data was assembled into a fasta file of 4 GB. The assembled genomic sequences consisted of 901,755 single sequence repeats (SSRs). In total 11,596 primer pairs were designed against these SSR motifs. The data generated provides baseline genomic information about the species and formulates a base for further sequencing of *A. pachyceras* through PACBio and HiC technologies. The novel developed SSR markers will facilitate genetic diversity and conservation studies for *Acacia* species.

## Specifications Table

**Table:** 

Subject	*Plant Sciences*
Specific subject area	*Genomics*
Type of data	*Tables, figures, raw sequencing reads, microsatellite motif file, SSR primer file*
How the data were acquired	Paired-end (2 × 150 cycles) sequencing on Illumina HiSeq 2500
Data format	Raw, analysed, filtered
Description of data collection	Fresh leaf samples were collected from the single specimen growing in the SANR area. DNA was isolated by CTAB method in triplicates. DNA purity and concentration were measured before sequencing. DNA sequences were obtained by Illumina HiSeq 2500 platform followed by *de novo* assembly using Platanus allee 2.0.
Data source location	• Institution: Kuwait Institute for Scientific Research • City/Town/Region: Shuwaikh, Kuwait • Country: Kuwait • Latitude and longitude (29°34′909″ N; 47°47′734″ E)
Data accessibility	Repository Name: National Centre for Biotechnology Information and figshare Data identification number: PRJNA754103 (SAMN20741683); https://doi.org/10.6084/m9.figshare.16745983; https://doi.org/10.6084/m9.figshare.16745974 Direct URL to data: https://www.ncbi.nlm.nih.gov/sra/SRX11728435 [*accn*] https://figshare.com/s/66481e64a92c148a5440 https://figshare.com/s/1875578cac289e3fd2bc

## Value of the Data

• This article provides the genome assembly of *Acacia pachyceras* Schwartz. and thus fills a gap of genomic studies in this genus.
• The genome assembly will be useful for geneticists interested in comparative genomics, conservation, breeding and phylogeny of *Acacia*.
• The genome assembly will serve as a reference for further high depth sequencing based on Pac Bio and Hi-C technologies.
• The information on SSR motifs and markers will be useful for the assessment of genetic diversity in this species.

## 1 Data Description

**Fig. 1:**
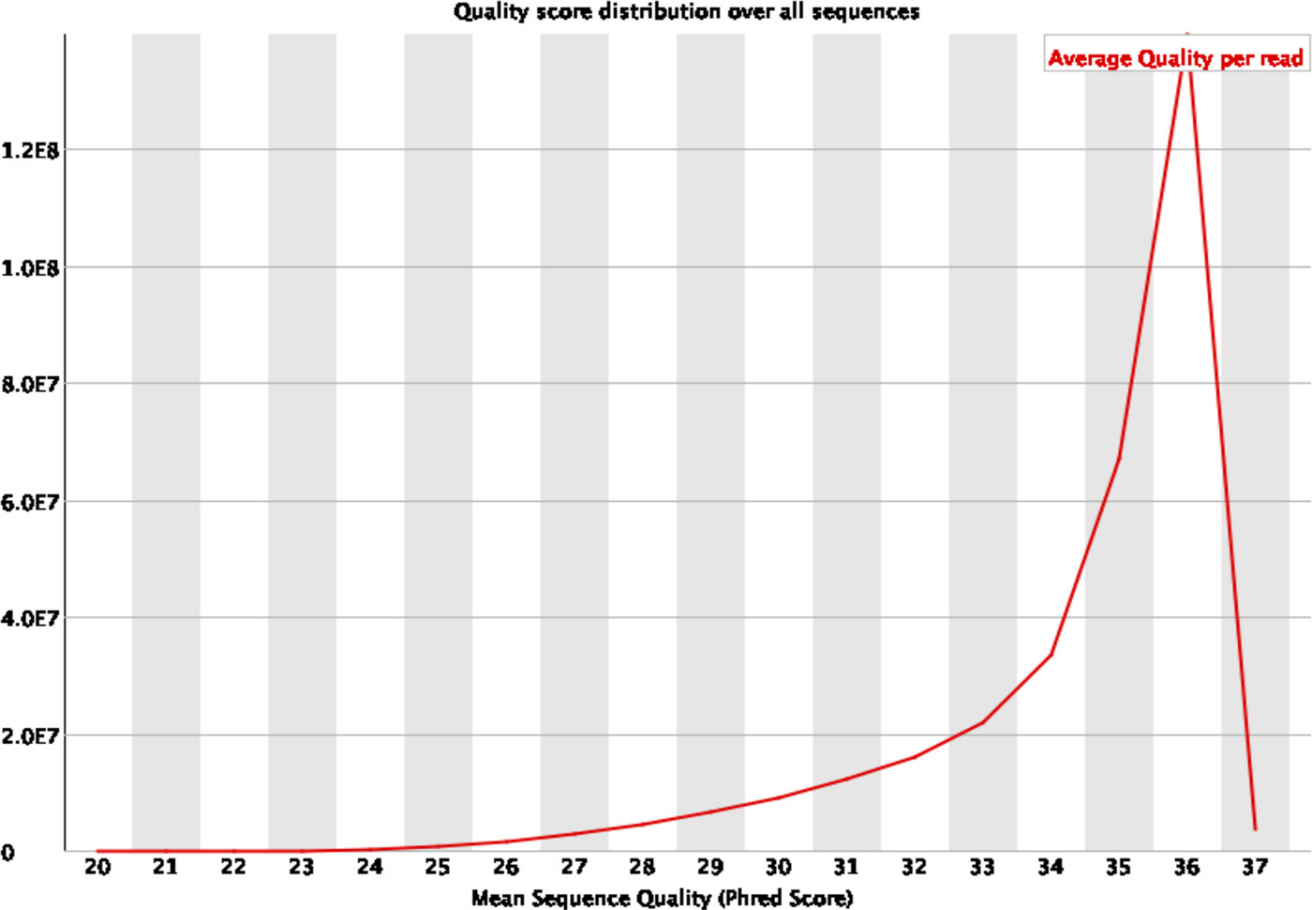
Sequence quality score. The x-axis represents the average Phred scores. The y-axis depicts the raw reads.

We present the data of genome survey of *Acacia pachyceras*, a woody tree of Leguminosae family through high-throughput sequencing [1], [2], [3]. Genome sequencing produced 108.90GB of raw data. After filtering the low quality reads total 96.79Gb data were used for further analysis (Table 1, Fig. 1). The sequencing data were deposited in the National Centre for Biotechnology Information (NCBI) short read archive (SRA) database under the accession number PRJNA754103 (SAMN20741683). The genome survey data provided a rough estimate of the genome size (700 Mb) of *A. pachyceras* through k-mer analysis. The raw sequences were *de novo* assembled into 4 Gb length. The assembled genome was used to filter single sequence repeats (SSR) motifs from the sequences. Primers flanking the SSR motifs were also designed that will aid towards genetic diversity studies of the species [4], [5], [6]. The data on SSR motifs and primers are available on figshare.

**Table 1:** Statistics of clean sequence data.

Raw Data (in Mb)	Insert Size (bp)	Read Length (bp)	Filtered Data (in Mb)

108,900	300–400	100;100	96,785

**Fig. 2:**
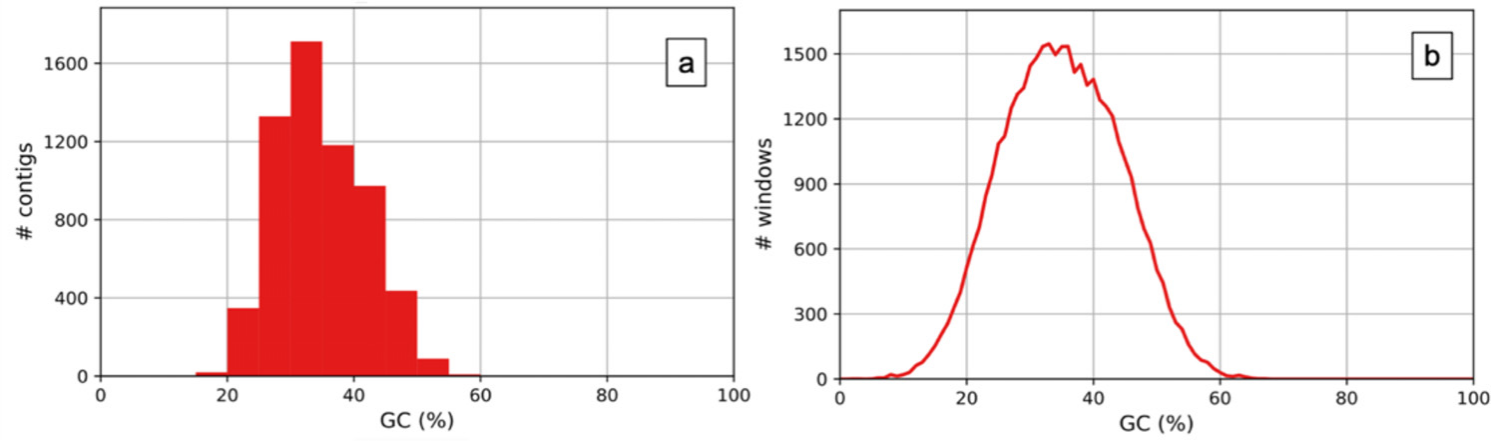
Guanine plus cytosine (GC) content analysis (a) The x-axis represents the GC content and the y-axis is the no of contigs. (b) The x-axis represents GC content and the y-axis is the no. of windows.

The assembly was constructed using the filtered reads which are approximately 87% of the total data. In total 51,761,594 contigs were generated with an N50 of 609. The largest contig was 3,140 bp in length. The total length of the contigs were 3,904,753 (Table 2). The GC content of the final draft assembly was 34.98% (Fig. 2a,b.)

**Table 2:** Basic statistics of genome assembly of Acacia pachyceras.

Statistics	PRJNA754103

# contigs (> = 0 bp)	51,761,594
# contigs (> = 1000 bp)	269
Total Length (> = 0 bp)	2,654,428,893
Total length ((> = 1000 bp)	330,734
# contigs	6,096
Largest contig	3,140
Total length	3,904,753
N50	609
N75	543
L50	2,514
L75	4,220

**Fig. 3:**
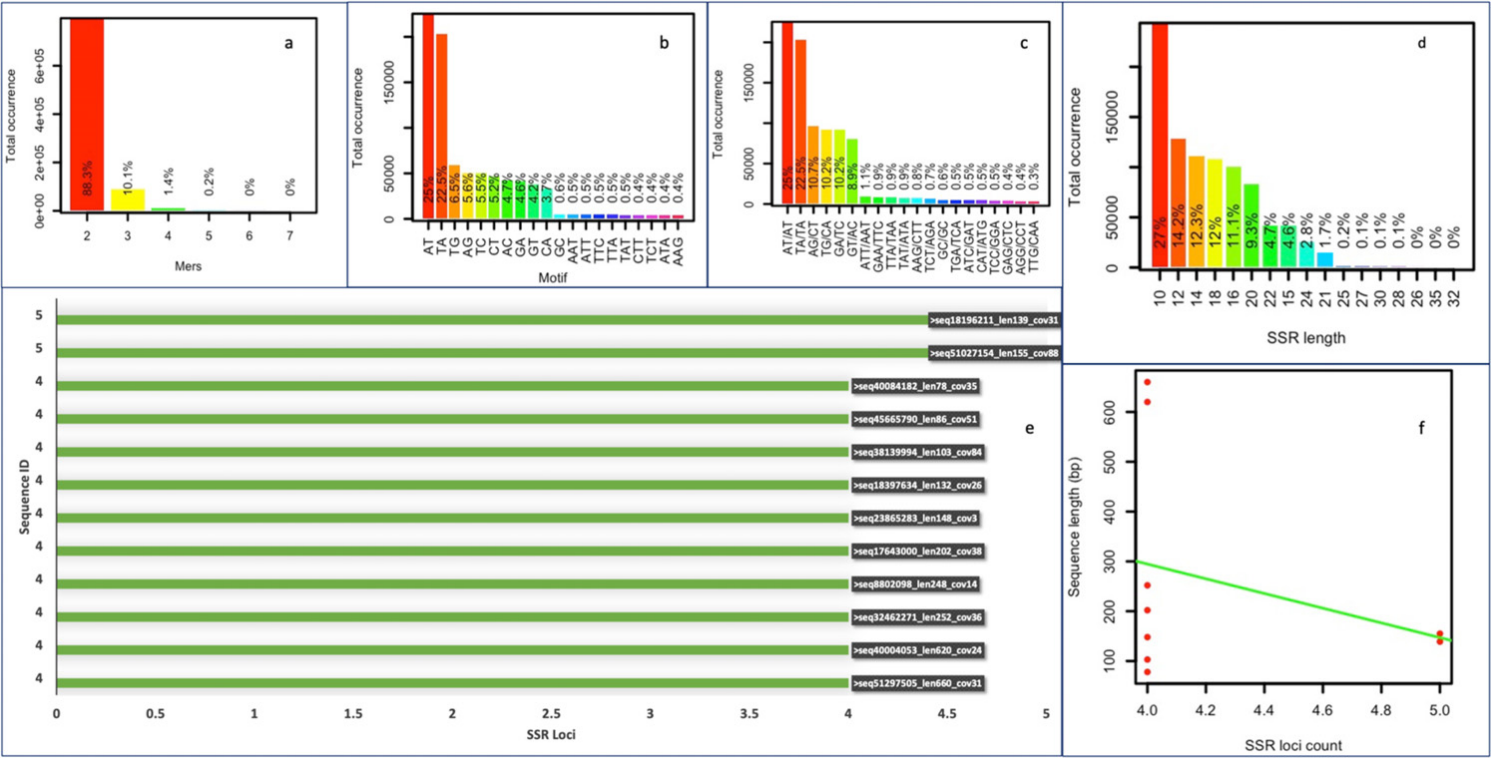
SSR motifs mined from genome assembly of *Acacia pachyceras* (a) Distribution of SSR motifs; (b) percentage of di and trinucleotides; (c) SSR length distribution; (d) Distribution of paired SSR motifs; (e) Contigs with the highest occurrence of SSRs; (f) SSR count versus sequence length.

Data mining of the whole genome sequences through GMATA yielded 901,755 SSR motifs that are listed in Table S1 (https://figshare.com/s/66481e64a92c148a5440). Further investigations on the motif types revealed, dinucleotides were the largest in number (796,441; 88%). These were followed by the trinucleotides (90,769; 10.06%), tetranucleotides (12,435; 1.3%), pentanucleotides (1651; 0.18%), and, hexanucleotides (418; 0.05%) (Fig. 3a). Frequency distribution of the repeat motifs were in the order of AT (25%) > TA (22.5%) > TG (6.5%) > AG (5.6%) >TC (5.5%) > CT (5.2%) > AC (4.7%) > GA (4.6%) > GT (4.2%) > CA (3.7%) and GC (0.6%) (Fig. 3b). All the tri- to hexanucleotides were below 1.0% in distribution. Among the paired dinucleotides, the highest number of SSR loci were AT/AT *ca.* 25% followed by TA/TA 22.5% > AG/CT, TG/CA, GA/TC, GT/AC *ca.* 10% (Fig. 3c). The number of SSR containing sequences decreased as the repeat number in motifs increased (Fig. 3d). The SSR loci length ranged from 10 to 32. The top contigs showing maximum no. of SSR loci are shown in Fig. 3e. The sequence length versus the SSR counts is presented in Fig. 3f. The primers designed against these SSR motifs are mentioned in Table S2 (https://figshare.com/s/1875578cac289e3fd2bc). In total 11,596 primer pairs were obtained from the 901,755 motifs. As these motifs were present on scaffolds, therefore, the motifs starting at the first 10–50 bp region or ending within the last 10–50 bp regions of the scaffolds could not have primers designed against them. In addition to this, certain motifs were situated at genomic regions that could not satisfy the specified primer designing parameters, and hence no primers were obtained for them as well. All the designed primers were 18 to 23 bp in size with annealing temperatures ranging between 57 and 62 °C. The GC content of the primers was between 30 and 70% and the final product length ranged amidst 100 to 400 bp.

## 2 Experimental Design, Materials and Methods

### 2.1 Plant material, DNA isolation, purity and yield

For the present investigation, fresh leaf samples from the single tree specimen located (29°34′909″ N, 47°47′734″ E) in the Sabah Al Nature Reserve (SANR) of Kuwait were snipped off the branches and immediately placed in polythene bags, appropriately labelled and transported on ice to the Kuwait Institute for Scientific Research (KISR) laboratories. The leaf tissue was weighed and ground to a fine powder in liquid nitrogen in an autoclaved mortar and pestle and stored at −20 °C until further use. The powdered sample was subjected to DNA extraction through the CTAB protocol [7,8]. DNA purity was checked through Nanodrop UV/Vis spectrophotometer (ThermoFisher, Waltham, MA). The DNA concentrations were estimated through the BR dsDNA assay (Qubit, Invitrogen, WA).

### 2.2 Next-generation sequencing

Sequencing libraries were prepared using the Nextera protocol (Illumina, San Diego, US). Approximately, 50 ng of DNA was subjected to enzymatic tagmentation followed by its amplification. Amplified libraries were purified using the AMPure XP beads (Beckmen and Coulter, Life Sciences, CA) The paired end-libraries were pooled and sequenced for 2 × 150 cycles on the Illumina HiSeq 2500 (Illumina, San Diego, US) platform. Quality parameters for the raw data were accessed through FASTQC v 0.119 [9]. Raw sequences were assembled by Platanus-allee 2.0 [10]. Trimmed sequences were first assembled into contigs and thereafter converted to scaffolds that were gap-closed to obtain a *.fa assembly. The assembly statistics were obtained through QUAST [11]. Genome size was predicted through k-mer analysis in JELLY-FISH 2.1.4 [12]. Microsatellite motifs were mined through GMATA 2.0 [13]. Primer 3 (v 3.0) was used to design primers against the filtered SSR motifs [14] applying the standard parameters (primer size: 18–23 bp; annealing temperature-57–62 °C; GC content-30–70%; final product size-100–400 bp).

## Ethics Statements

Not applicable.

## CRediT authorship contribution statement

**Nazima Habibi:** Conceptualization, Writing – original draft, Writing – review & editing. **Fadila Al Salameen:** Supervision, Resources. **Muhammed Rahman:** Conceptualization, Writing – review & editing. **Vinod Kumar:** Formal analysis. **Sami Al Amad:** Methodology. **Anisha Shajan:** Methodology. **Farhana Zakir:** Methodology. **Nasreem Abdul Razzack:** Methodology. **Waiel Hussain Tinwala:** Software, Data curation, Visualization.

## Declaration of Competing Interest

The authors declare that they have no known competing financial interests or personal relationships that could have appeared to influence the work reported in this paper.
